# 2,2′,5′,6-Tetra­chloro-4-[(1*S*)-1-methyl­prop­oxy]biphen­yl

**DOI:** 10.1107/S1600536813014098

**Published:** 2013-05-31

**Authors:** Hans-Joachim Lehmler, Huimin Wu, Sean Parkin

**Affiliations:** a The University of Iowa, Department of Occupational and Environmental Health, Iowa City, IA 52242-5000, USA; b University of Kentucky, Department of Chemistry, Lexington, KY 40506-0055, USA

## Abstract

In the title mol­ecule, C_16_H_14_Cl_4_O, the dihedral angle between the least-square planes of the benzene rings is 84.40 (7)°. No unusual intermolecular interactions are present.

## Related literature

For related literature about polychlorinated bi­phenyls, see: Lehmler *et al.* (2010 ▶); Warner *et al.* (2009 ▶). For crystal structures of PCB derivatives with two or less *ortho* chlorine substituents, see: Mannila & Rissanen (1994 ▶); Miao *et al.* (1996 ▶); Rissanen *et al.* (1988*a* ▶); Shaikh *et al.* (2008 ▶); Singh *et al.* (1986 ▶); van der Sluis *et al.* (1990 ▶); Vyas *et al.* (2006 ▶). For crystal structures of PCB derivatives with three *ortho* chlorine substituents, see: Lehmler *et al.* (2005 ▶); Rissanen *et al.* (1988*b* ▶). For crystal structures of PCB derivatives with four *ortho* chlorine substituents, see: Pedersen (1975 ▶); Singh & McKinney (1979 ▶). For literature about the Mitsunobu reaction, see: Fujita *et al.* (2001 ▶).
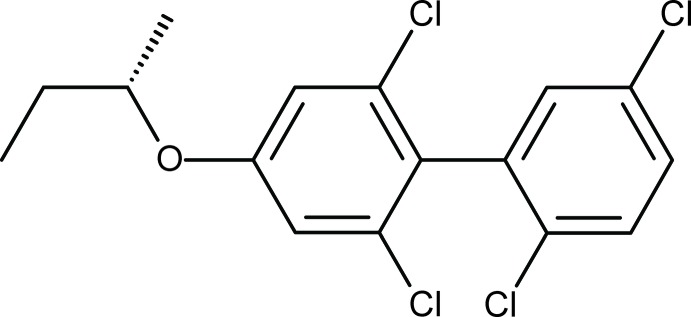


## Experimental

### 

#### Crystal data

C_16_H_14_Cl_4_O
*M* *_r_* = 364.07
Orthorhombic, 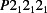

*a* = 10.3301 (2) Å
*b* = 10.5415 (2) Å
*c* = 15.2160 (3) Å
*V* = 1656.94 (6) Å^3^
*Z* = 4
Mo *K*α radiation
μ = 0.71 mm^−1^
*T* = 90 K
0.25 × 0.25 × 0.08 mm

#### Data collection

Nonius KappaCCD diffractometer
Absorption correction: multi-scan (*SCALEPACK*; Otwinowski & Minor, 1997 ▶) *T*_min_ = 0.843, *T*_max_ = 0.946
22321 measured reflections
3797 independent reflections
3351 reflections with *I* > 2σ(*I*)
*R*_int_ = 0.053

#### Refinement

*R*[*F* ^2^ > 2σ(*F* ^2^)] = 0.034
*wR*(*F* ^2^) = 0.081
*S* = 1.09
3797 reflections
192 parameters
H-atom parameters constrained
Δρ_max_ = 0.30 e Å^−3^
Δρ_min_ = −0.28 e Å^−3^
Absolute structure: Flack (1983 ▶), 1625 Friedel pairs
Flack parameter: 0.00 (6)

### 

Data collection: *COLLECT* (Nonius, 1998 ▶); cell refinement: *SCALEPACK* (Otwinowski & Minor, 1997 ▶); data reduction: *DENZO-SMN* (Otwinowski & Minor, 1997 ▶); program(s) used to solve structure: *SHELXS97* (Sheldrick, 2008 ▶); program(s) used to refine structure: *SHELXL97* (Sheldrick, 2008 ▶); molecular graphics: *XP* in *SHELXTL* (Sheldrick, 2008 ▶); software used to prepare material for publication: *SHELXL97* and local procedures.

## Supplementary Material

Crystal structure: contains datablock(s) I, global. DOI: 10.1107/S1600536813014098/ng5329sup1.cif

Structure factors: contains datablock(s) I. DOI: 10.1107/S1600536813014098/ng5329Isup2.hkl

Supplementary material file. DOI: 10.1107/S1600536813014098/ng5329Isup3.cml

Additional supplementary materials:  crystallographic information; 3D view; checkCIF report

## Figures and Tables

**Fig. 1.**
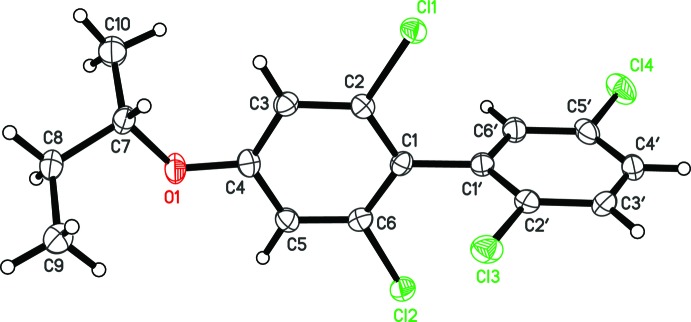
View of the title compound showing the atom-labeling scheme. Displacement ellipsoids are drawn at the 50% probability level.

## supplementary crystallographic information

### Comment

The title compound was synthesized as an intermediate in ongoing efforts to synthesize atropisomerically pure hydroxylated polychlorinated biphenyls (PCBs) for metabolism and toxicological studies (Lehmler *et al.*, 2010; Warner *et al.*, 2009). The dihedral angle between the two phenyl rings of the title compound, an important determinant of the toxicity of PCBs, was 84.40 (7)°. Comparable solid state dihedral (82–83°) have been reported for structurally related PCB derivatives with three *ortho* chlorine substituents (Lehmler *et al.*, 2005; Rissanen *et al.*, 1988*b*). Slightly larger (84–87°) dihedral angles have been observed for PCB derivatives with four *ortho* chlorine substituents (Pedersen, 1975; Singh & McKinney, 1979). Smaller solid state dihedral angles have been reported for PCB derivatives with zero, one or two *ortho* chlorine substituents due to the smaller steric demand of multiple hydrogen substituents in *ortho* position (Mannila & Rissanen, 1994; Miao *et al.*, 1996; Rissanen *et al.*, 1988*a*; Shaikh *et al.*, 2008; Singh *et al.*, 1986; van der Sluis *et al.*, 1990; Vyas *et al.*, 2006).

### Experimental

The title compound was synthesized by the Mitsunobu reaction of 2,2',5',6-tetrachloro-biphenyl-4-ol with (*R*)-isobutanol in THF (Fujita *et al.*, 2001). Crystals suitable for crystal structure analysis were obtained by slowly evaporating a methanolic solution of the title compound.

### Refinement

H atoms were found in difference Fourier maps and subsequently placed in idealized positions with constrained distances of 0.98 Å (RCH_3_), 0.99 Å (*R*_2_CH_2_), 1.00 Å (*R*_3_CH), 0.95 Å (C_sp2_H), and with *U*_iso_(H) values set to either 1.2*U*_eq_ or 1.5*U*_eq_ (RCH_3_) of the attached atom. The absolute configuration was determined from 1625 Friedel pairs [Flack '*x*' = 0.00 (6)].

### Crystal data

**Table:**

C_16_H_14_Cl_4_O	*F*(000) = 744
*M**_r_* = 364.07	*D*_x_ = 1.459 Mg m^−^^3^
Orthorhombic, *P*2_1_2_1_2_1_	Mo *K*α radiation, λ = 0.71073 Å
Hall symbol: P 2ac 2ab	Cell parameters from 2184 reflections
*a* = 10.3301 (2) Å	θ = 1.0–27.5°
*b* = 10.5415 (2) Å	µ = 0.71 mm^−^^1^
*c* = 15.2160 (3) Å	*T* = 90 K
*V* = 1656.94 (6) Å^3^	Plate, colourless
*Z* = 4	0.25 × 0.25 × 0.08 mm

### Data collection

**Table:**

Nonius KappaCCD diffractometer	3797 independent reflections
Radiation source: fine-focus sealed tube	3351 reflections with *I* > 2σ(*I*)
Graphite monochromator	*R*_int_ = 0.053
Detector resolution: 9.1 pixels mm^-1^	θ_max_ = 27.5°, θ_min_ = 2.4°
ω scans at fixed χ = 55°	*h* = −13→13
Absorption correction: multi-scan (*SCALEPACK*; Otwinowski & Minor, 1997)	*k* = −13→13
*T*_min_ = 0.843, *T*_max_ = 0.946	*l* = −19→19
22321 measured reflections	

### Refinement

**Table:**

Refinement on *F*^2^	Secondary atom site location: difference Fourier map
Least-squares matrix: full	Hydrogen site location: inferred from neighbouring sites
*R*[*F*^2^ > 2σ(*F*^2^)] = 0.034	H-atom parameters constrained
*wR*(*F*^2^) = 0.081	*w* = 1/[σ^2^(*F*_o_^2^) + (0.0401*P*)^2^ + 0.5301*P*] where *P* = (*F*_o_^2^ + 2*F*_c_^2^)/3
*S* = 1.09	(Δ/σ)_max_ < 0.001
3797 reflections	Δρ_max_ = 0.30 e Å^−^^3^
192 parameters	Δρ_min_ = −0.28 e Å^−^^3^
0 restraints	Absolute structure: Flack (1983), 1625 Friedel pairs
Primary atom site location: structure-invariant direct methods	Flack parameter: 0.00 (6)

### Special details

**Table:**

Geometry. All e.s.d.'s (except the e.s.d. in the dihedral angle between two l.s. planes) are estimated using the full covariance matrix. The cell e.s.d.'s are taken into account individually in the estimation of e.s.d.'s in distances, angles and torsion angles; correlations between e.s.d.'s in cell parameters are only used when they are defined by crystal symmetry. An approximate (isotropic) treatment of cell e.s.d.'s is used for estimating e.s.d.'s involving l.s. planes.
Refinement. Refinement of *F*^2^ against all reflections. The weighted *R*-value *wR* and goodness of fit *S* are based on *F*^2^. Conventional *R*-values *R* are based on *F*, with *F* set to zero for negative *F*^2^. The threshold expression of *F*^2^ > 2σ(*F*^2^) is used only for calculating *R*-factors(gt) *etc*. and is not relevant to the choice of reflections for refinement. *R*-values based on *F*^2^ are statistically about twice as large as those based on *F*, and *R*-values based on ALL data will be even larger.

### Fractional atomic coordinates and isotropic or equivalent isotropic displacement parameters (Å^2^)

**Table:**

	*x*	*y*	*z*	*U*_iso_*/*U*_eq_	
O1	0.09110 (15)	0.46820 (16)	0.13102 (10)	0.0267 (4)	
Cl1	0.32228 (5)	0.50012 (5)	0.42961 (3)	0.02188 (13)	
Cl2	0.53731 (6)	0.28777 (6)	0.14184 (4)	0.02615 (14)	
Cl3	0.44229 (6)	0.14044 (5)	0.36487 (4)	0.02755 (14)	
Cl4	0.86539 (7)	0.55656 (7)	0.40389 (4)	0.03806 (18)	
C1'	0.5425 (2)	0.3734 (2)	0.33248 (14)	0.0207 (5)	
C1	0.4219 (2)	0.3999 (2)	0.28166 (14)	0.0188 (5)	
C2	0.3142 (2)	0.4581 (2)	0.31899 (14)	0.0185 (4)	
C3	0.2007 (2)	0.4834 (2)	0.27365 (14)	0.0206 (5)	
H3	0.1296	0.5237	0.3018	0.025*	
C4	0.1940 (2)	0.4478 (2)	0.18498 (15)	0.0215 (5)	
C5	0.2990 (2)	0.3874 (2)	0.14558 (15)	0.0217 (5)	
H5	0.2945	0.3625	0.0857	0.026*	
C6	0.4089 (2)	0.3641 (2)	0.19355 (14)	0.0194 (5)	
C7	−0.0337 (2)	0.5044 (2)	0.16686 (15)	0.0243 (5)	
H7	−0.0453	0.4652	0.2262	0.029*	
C8	−0.1328 (2)	0.4502 (2)	0.10372 (16)	0.0274 (5)	
H8A	−0.1179	0.4871	0.0447	0.033*	
H8B	−0.2203	0.4764	0.1232	0.033*	
C9	−0.1291 (3)	0.3067 (3)	0.0965 (2)	0.0373 (7)	
H9A	−0.0428	0.2797	0.0773	0.056*	
H9B	−0.1938	0.2785	0.0536	0.056*	
H9C	−0.1484	0.2691	0.1539	0.056*	
C10	−0.0439 (3)	0.6479 (2)	0.17430 (17)	0.0325 (6)	
H10A	0.0290	0.6802	0.2089	0.049*	
H10B	−0.1254	0.6703	0.2034	0.049*	
H10C	−0.0419	0.6855	0.1154	0.049*	
C2'	0.5617 (2)	0.2565 (2)	0.37208 (15)	0.0235 (5)	
C3'	0.6732 (3)	0.2293 (2)	0.41872 (16)	0.0303 (6)	
H3'	0.6846	0.1480	0.4446	0.036*	
C4'	0.7682 (3)	0.3210 (3)	0.42750 (17)	0.0315 (6)	
H4'	0.8454	0.3033	0.4592	0.038*	
C5'	0.7492 (2)	0.4387 (3)	0.38960 (15)	0.0265 (5)	
C6'	0.6381 (2)	0.4661 (2)	0.34183 (15)	0.0232 (5)	
H6'	0.6272	0.5472	0.3157	0.028*	

### Atomic displacement parameters (Å^2^)

**Table:**

	*U*^11^	*U*^22^	*U*^33^	*U*^12^	*U*^13^	*U*^23^
O1	0.0178 (8)	0.0421 (11)	0.0203 (8)	0.0042 (7)	−0.0031 (7)	−0.0005 (8)
Cl1	0.0234 (3)	0.0254 (3)	0.0169 (2)	0.0005 (2)	0.0008 (2)	−0.0006 (2)
Cl2	0.0243 (3)	0.0305 (3)	0.0237 (3)	0.0061 (2)	0.0010 (2)	−0.0041 (2)
Cl3	0.0366 (3)	0.0196 (3)	0.0264 (3)	−0.0011 (2)	−0.0004 (3)	0.0009 (2)
Cl4	0.0238 (3)	0.0563 (4)	0.0341 (3)	−0.0085 (3)	−0.0017 (3)	−0.0120 (3)
C1'	0.0194 (12)	0.0243 (11)	0.0182 (10)	0.0029 (10)	0.0008 (9)	−0.0018 (9)
C1	0.0194 (12)	0.0159 (10)	0.0212 (11)	0.0010 (9)	−0.0003 (9)	0.0032 (8)
C2	0.0201 (12)	0.0185 (10)	0.0168 (10)	−0.0016 (9)	0.0004 (9)	0.0018 (9)
C3	0.0198 (12)	0.0215 (12)	0.0205 (10)	0.0017 (9)	0.0020 (9)	−0.0004 (9)
C4	0.0194 (12)	0.0240 (11)	0.0209 (11)	−0.0024 (10)	−0.0016 (9)	0.0032 (10)
C5	0.0230 (12)	0.0238 (11)	0.0182 (11)	−0.0001 (9)	−0.0013 (10)	−0.0013 (9)
C6	0.0174 (11)	0.0185 (11)	0.0223 (11)	−0.0011 (9)	0.0040 (9)	0.0004 (9)
C7	0.0178 (12)	0.0292 (11)	0.0258 (11)	0.0039 (10)	−0.0020 (9)	−0.0002 (10)
C8	0.0209 (12)	0.0294 (13)	0.0319 (12)	0.0032 (11)	−0.0061 (11)	−0.0004 (11)
C9	0.0304 (14)	0.0307 (14)	0.0507 (17)	0.0060 (12)	−0.0084 (14)	−0.0089 (13)
C10	0.0313 (14)	0.0272 (13)	0.0388 (14)	0.0007 (12)	−0.0068 (12)	−0.0012 (11)
C2'	0.0278 (13)	0.0237 (11)	0.0189 (11)	0.0045 (10)	−0.0021 (10)	−0.0035 (9)
C3'	0.0382 (15)	0.0269 (13)	0.0258 (12)	0.0154 (11)	−0.0072 (11)	−0.0039 (10)
C4'	0.0281 (14)	0.0423 (16)	0.0243 (12)	0.0143 (12)	−0.0086 (11)	−0.0104 (11)
C5'	0.0168 (12)	0.0398 (14)	0.0228 (12)	−0.0010 (11)	−0.0001 (10)	−0.0090 (11)
C6'	0.0213 (12)	0.0274 (12)	0.0210 (11)	0.0013 (10)	0.0000 (9)	−0.0008 (9)

### Geometric parameters (Å, º)

**Table:**

O1—C4	1.360 (3)	C7—C10	1.520 (3)
O1—C7	1.451 (3)	C7—H7	1.0000
Cl1—C2	1.743 (2)	C8—C9	1.518 (4)
Cl2—C6	1.739 (2)	C8—H8A	0.9900
Cl3—C2'	1.740 (3)	C8—H8B	0.9900
Cl4—C5'	1.741 (3)	C9—H9A	0.9800
C1'—C2'	1.386 (3)	C9—H9B	0.9800
C1'—C6'	1.396 (3)	C9—H9C	0.9800
C1'—C1	1.493 (3)	C10—H10A	0.9800
C1—C2	1.391 (3)	C10—H10B	0.9800
C1—C6	1.399 (3)	C10—H10C	0.9800
C2—C3	1.386 (3)	C2'—C3'	1.383 (3)
C3—C4	1.402 (3)	C3'—C4'	1.384 (4)
C3—H3	0.9500	C3'—H3'	0.9500
C4—C5	1.393 (3)	C4'—C5'	1.382 (4)
C5—C6	1.372 (3)	C4'—H4'	0.9500
C5—H5	0.9500	C5'—C6'	1.389 (3)
C7—C8	1.515 (3)	C6'—H6'	0.9500
			
C4—O1—C7	120.62 (17)	C7—C8—H8B	108.8
C2'—C1'—C6'	118.5 (2)	C9—C8—H8B	108.8
C2'—C1'—C1	120.7 (2)	H8A—C8—H8B	107.7
C6'—C1'—C1	120.8 (2)	C8—C9—H9A	109.5
C2—C1—C6	115.7 (2)	C8—C9—H9B	109.5
C2—C1—C1'	122.57 (19)	H9A—C9—H9B	109.5
C6—C1—C1'	121.7 (2)	C8—C9—H9C	109.5
C3—C2—C1	123.9 (2)	H9A—C9—H9C	109.5
C3—C2—Cl1	118.18 (17)	H9B—C9—H9C	109.5
C1—C2—Cl1	117.91 (17)	C7—C10—H10A	109.5
C2—C3—C4	118.0 (2)	C7—C10—H10B	109.5
C2—C3—H3	121.0	H10A—C10—H10B	109.5
C4—C3—H3	121.0	C7—C10—H10C	109.5
O1—C4—C5	114.89 (19)	H10A—C10—H10C	109.5
O1—C4—C3	125.2 (2)	H10B—C10—H10C	109.5
C5—C4—C3	119.9 (2)	C3'—C2'—C1'	121.8 (2)
C6—C5—C4	119.8 (2)	C3'—C2'—Cl3	118.48 (18)
C6—C5—H5	120.1	C1'—C2'—Cl3	119.76 (18)
C4—C5—H5	120.1	C2'—C3'—C4'	119.7 (2)
C5—C6—C1	122.7 (2)	C2'—C3'—H3'	120.2
C5—C6—Cl2	118.25 (17)	C4'—C3'—H3'	120.2
C1—C6—Cl2	119.03 (18)	C5'—C4'—C3'	119.1 (2)
O1—C7—C8	105.22 (18)	C5'—C4'—H4'	120.4
O1—C7—C10	110.6 (2)	C3'—C4'—H4'	120.4
C8—C7—C10	112.1 (2)	C4'—C5'—C6'	121.5 (2)
O1—C7—H7	109.6	C4'—C5'—Cl4	119.38 (19)
C8—C7—H7	109.6	C6'—C5'—Cl4	119.1 (2)
C10—C7—H7	109.6	C5'—C6'—C1'	119.5 (2)
C7—C8—C9	113.9 (2)	C5'—C6'—H6'	120.3
C7—C8—H8A	108.8	C1'—C6'—H6'	120.3
C9—C8—H8A	108.8		
			
C2'—C1'—C1—C2	94.3 (3)	C2—C1—C6—Cl2	−178.73 (16)
C6'—C1'—C1—C2	−85.2 (3)	C1'—C1—C6—Cl2	−0.5 (3)
C2'—C1'—C1—C6	−83.9 (3)	C4—O1—C7—C8	−149.7 (2)
C6'—C1'—C1—C6	96.7 (3)	C4—O1—C7—C10	89.1 (3)
C6—C1—C2—C3	−1.5 (3)	O1—C7—C8—C9	61.7 (3)
C1'—C1—C2—C3	−179.7 (2)	C10—C7—C8—C9	−178.1 (2)
C6—C1—C2—Cl1	177.90 (17)	C6'—C1'—C2'—C3'	−1.2 (3)
C1'—C1—C2—Cl1	−0.4 (3)	C1—C1'—C2'—C3'	179.3 (2)
C1—C2—C3—C4	0.2 (3)	C6'—C1'—C2'—Cl3	177.88 (16)
Cl1—C2—C3—C4	−179.12 (18)	C1—C1'—C2'—Cl3	−1.6 (3)
C7—O1—C4—C5	166.6 (2)	C1'—C2'—C3'—C4'	0.9 (4)
C7—O1—C4—C3	−14.0 (3)	Cl3—C2'—C3'—C4'	−178.25 (19)
C2—C3—C4—O1	−178.5 (2)	C2'—C3'—C4'—C5'	0.3 (4)
C2—C3—C4—C5	0.8 (3)	C3'—C4'—C5'—C6'	−1.0 (4)
O1—C4—C5—C6	178.9 (2)	C3'—C4'—C5'—Cl4	177.56 (19)
C3—C4—C5—C6	−0.6 (3)	C4'—C5'—C6'—C1'	0.7 (3)
C4—C5—C6—C1	−0.8 (3)	Cl4—C5'—C6'—C1'	−177.93 (17)
C4—C5—C6—Cl2	179.69 (18)	C2'—C1'—C6'—C5'	0.4 (3)
C2—C1—C6—C5	1.7 (3)	C1—C1'—C6'—C5'	179.9 (2)
C1'—C1—C6—C5	−180.0 (2)		
